# Comparison of experimental and radiobiological model-predicted survival of human laryngeal squamous cell carcinoma cells in a helium spread-out Bragg peak

**DOI:** 10.1016/j.phro.2026.101047

**Published:** 2026-07-28

**Authors:** Thomas Berger, Gersende Alphonse, Mario Alcocer-Avila, Caterina Monini, Charbel Koumeir, Claire Rodriguez-Lafrasse, Flavien Ralite, Nicolas Varmenot, Noel Servagent, Vincent Métivier, Ferid Haddad, Alexis Pereda, Lydia Maigne, Jean Michel Létang, Étienne Testa, Michaël Beuve

**Affiliations:** a Universite Claude Bernard Lyon 1, CNRS/IN2P3, IP2I Lyon, UMR 5822, F-69100 Villeurbanne, France; b Radiation Oncology Department, Center Hospitalier Lyon Sud, Hospices Civils de Lyon, Pierre Bénite, France; c Department of Biochemistry and Molecular Biology, Lyon-Sud Hospital, Hospices Civils de Lyon, Pierre-Bénite, France; d Laboratoire SUBATECH, UMR 6457 CNRS-IN2P3, IMT Atlantique, Nantes Université, France; e GIP ARRONAX, Saint Herblain, France; f INSERM, U1232, CRCI^2^NA, Nantes, France; g Institut de Cancérologie de l'Ouest, F-44800, Saint-Herblain, France; h Laboratoire de Physique de Clermont, Université Clermont Auvergne, CNRS/IN2P3, 4 Avenue Blaise Pascal, 63178 Aubière, France; i INSA-Lyon, Université Claude Bernard Lyon 1, CREATIS, CNRS UMR5220, Inserm U1294, 69373 Lyon, France

**Keywords:** Monte Carlo simulation, surviving fraction, SQ20B, Helium therapy, NanOx

## Abstract

**Background and Purpose:**

Helium ion therapy offers favorable dose distributions and potential biological advantages over photon and proton therapies, yet data on cellular response in clinically relevant Spread-Out Bragg Peak (SOBP) configurations remain limited. This study measured the surviving fraction of radioresistant human laryngeal squamous cell carcinoma (SQ20B) cells irradiated in a helium SOBP and compared experimental results with predictions from NanOx, a nanodosimetry and oxidative stress model implemented in Monte Carlo simulations.

**Materials and Methods:**

SQ20B cells were irradiated at three positions along a helium SOBP using a passive beamline. Survival fractions were measured for doses between 1 and 4 Gy. The setup was simulated in GATE, and survival was predicted using the NanOx model via the Biodose Actor. Relative biological effectiveness (RBE) was derived from linear quadratic fits using photon reference parameters.

**Results:**

NanOx predicted decreasing survival towards the distal edge, with surviving fractions at 4 Gy of 11%, 7%, and 4% at the proximal edge, plateau, and distal edge, respectively. Experimentally, fitted surviving fractions at 4 Gy were 10%, 12%, and 8%, showing no spatial trend. Deviations between model predictions and experimental averages ranged from 20 to 37%. Experimental RBE values ranged from 2.1 to 2.4, in reasonable agreement with NanOx predictions (2.1–2.9).

**Conclusions:**

In this SQ20B cell model under the present experimental conditions, NanOx predictions differed from experimental data by 20–37% depending on position. These results support further validation of the model in a broader range of cell lines and helium ion irradiation configurations.

## Highlights

• Survival of laryngeal carcinoma cells was measured in a helium ion beam.
• Helium ions showed enhanced biological effectiveness in cell experiments.
• Experimental survival was compared with radiobiological model predictions.
• Predicted and measured survival showed partial agreement.

## Graphical abstract

**Figure:**
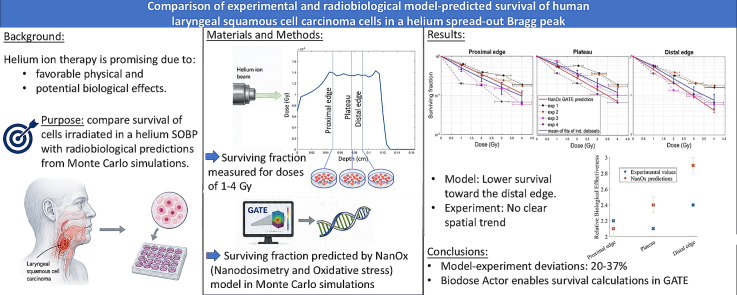

## 1 Introduction

Particle therapy allows for more conformal dose distributions compared to conventional photon therapy. Over the past three decades, the two main hadrontherapy modalities employed clinically used either protons or carbon ions to deliver dose to tumoral regions. Helium ions recently re-gained traction as a promising alternative after passive scattering helium ion therapy stopped being used at the Lawrence Berkeley National Laboratory in the 1990s [1], [2], [3], [4]. An illustration of this recent interest is the first clinical implementation of raster-scanned helium ion beam therapy in 2023 at the Heidelberg Ion Beam Therapy Center (HIT) [1]. This renewed interest can partly be explained by helium ions' physical properties that are midway between those of protons and carbon ions. Indeed, they have a reduced range straggling and lateral scattering compared to protons and a reduced fragmentation tail compared to carbon ions [5], [6], [7]. These characteristics offer the possibility to form dose distributions with steep dose gradients which may be used to further spare the healthy tissues.

In order to exploit these favorable physical characteristics, the radiobiological response to helium ion irradiation needs to be determined and modeled. For carbon ions, the Relative Biological Effectiveness (RBE) is estimated using radiobiological models already incorporated in Treatment Planning Systems: the Local Effect Model (LEM) [8], [9], [10], [11] and the Microdosimetric Kinetic Model (MKM) [12], [13]. Several alternative models were recently developed for hadrontherapy, one of which is called NanOx for Nano-dosimetry and Oxidative stress [14], [15], [16], [17]. This model considers the stochastic nature of energy deposition at both the nanometric and microscopic scales. In its theoretical framework, cellular inactivation may be the consequence of either local lethal events, or global events. The former are caused by one track and described in terms of nano-dosimetry, while the latter result from combined deleterious effects and are attributed to sublethal damage and oxidative stress. The results from a study previously published by our research group [18], indicate that NanOx accurately predicts survival curves of biological samples irradiated by monoenergetic ions.

In order to achieve precise external beam helium therapy treatment planning, a broad and diligent analysis of its biological effect is required. On this topic, the literature remains relatively scarce with a study by Mairani et al. [19] finding in 2016 a total of seventeen articles published between 1977 and 2011 on about 20 different cell lines. Fewer studies have investigated the biological response to helium irradiation in a Spread Out Bragg Peak (SOBP) configuration while this is of particular relevance as it represents a clinical-like scenario. Our study aims at contributing to this gap in the literature by assessing the response of human laryngeal squamous cell carcinoma (SQ20B) cells, a radioresistant head and neck squamous cell carcinoma (HNSCC) model, to helium SOBP irradiation, therefore in mixed field conditions. This work should be considered an initial radiobiological characterization study in a single cell line model rather than a general assessment of tumour response to helium ions.

GATE (Geant4 Application for Tomographic Emission) is an open-source Monte Carlo simulation platform [20], [21] based on GEANT4 (GEometry ANd Tracking 4), a toolkit for particle transport simulation and interactions in matter. It was specifically developed for radiation therapy and dosimetry applications and is therefore a particularly useful tool for simulating such irradiation experiments. Using GATE is of particular relevance in this context as it can now be used to determine directly the surviving fraction of irradiated cells as predicted by the NanOx radiobiological model [22].

The purpose of this work was to measure survival curves, for SQ20B cells irradiated in a helium SOBP, and compare them to values predicted by the NanOx model in the GATE Monte Carlo simulation platform.

## 2 Materials and methods

### 2.1 Experimental device

**Fig. 1:**
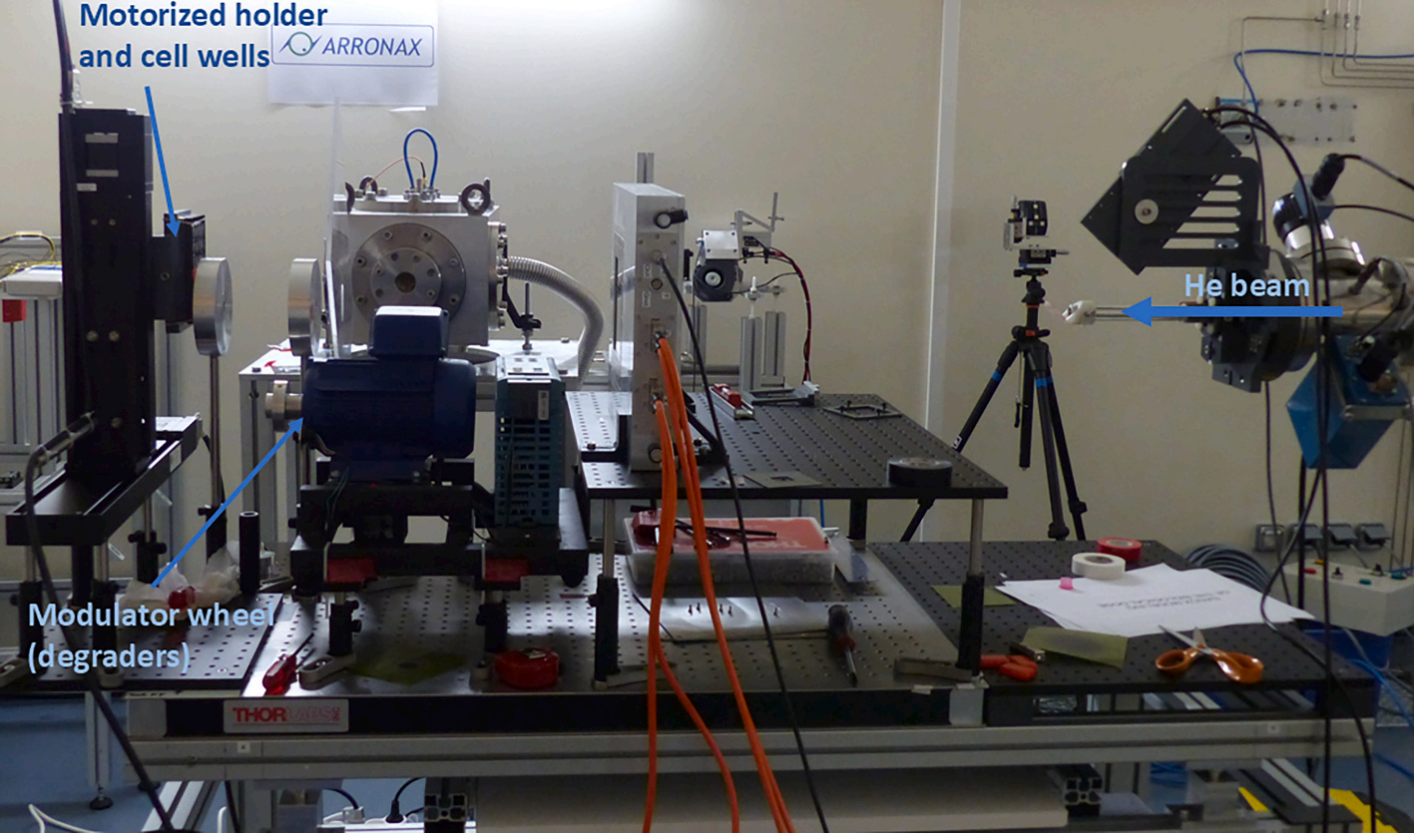
Photograph of the experimental beamline used at the ARRONAX cyclotron facility for helium SOBP irradiation of SQ20B cells.

A beamline enabling the passive irradiation of biological samples in a helium SOBP was set up at the ARRONAX (Accelerator for Research in Radiochemistry and Oncology at Nantes Atlantique) cyclotron facility [23]. This experimental device, shown in Fig. 1 and described in detail in Supplementary Material A, is composed of one diffuser, two collimators and a range-modulating wheel made of seven sectors of aluminium. This rotating element degrades the energy of the incident 16.9 MeV/u helium beam creating eight pristine Bragg peaks which, when combined, form an SOBP. The thickness and weights of the sectors of the range-modulating wheel are specified in Supplementary Material B. The depth of the distal edge of this SOBP is approximately 1.5 mm in water. The simulated SOBP was validated experimentally by irradiating a stack of six radio-chromic films (HDV2). Film response was analyzed to assess SOBP extension and homogeneity. The potential LET dependence of the film response, which may affect absolute dose accuracy, was not corrected for and is considered a limitation of this validation approach. The dose was indirectly quantified through the online detection of photons emitted by the interaction of the incident ions with a 2 μm thick copper foil. The mean dose rate was 3 Gy/min.

### 2.2 GATE Monte Carlo simulation

**Fig. 2:**
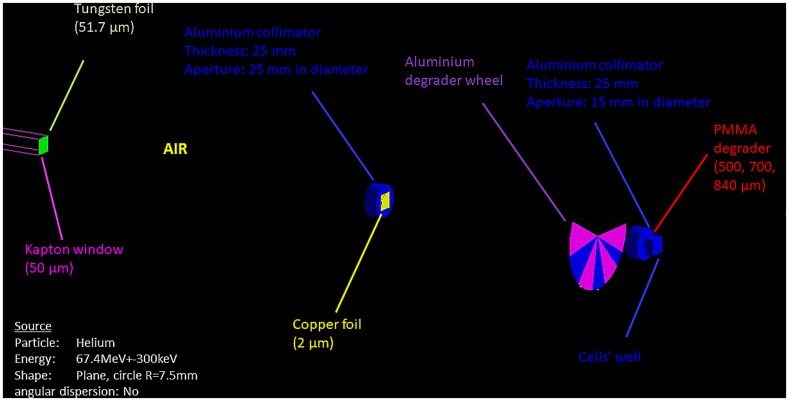
Geometrical model of the experimental beamline implemented in the GATE Monte Carlo simulation for dose and biological dose calculations.

The beamline geometry was numerically reproduced in GATE v9.4 as shown in Fig. 2. The Monte Carlo simulations were run using the QGSP_BIC_EMZ physics list as it accurately models both hadronic and electromagnetic interactions in our energy range. All relevant information on the simulation is detailed in Supplementary Material A in accordance with [24].

### 2.3 NanOx parameters

The parameters of the NanOx model were tuned based on experimental values of the alpha coefficient for protons and carbon ions of different energies [25], [26]. This procedure involves the optimization of the NanOx effective local lethal function (ELLF) which is related to the contribution of local lethal events to cell survival. This function characterizes the response of the cell line of interest by means of three free parameters: h, z_0_ and sigma [26], [27], as described in the following equation:

$$F\left(z\right)=\frac{h}{2}\left[1+\mathrm{erf}\left(\frac{z-z_{0}}{\sigma }\right)\right]$$

The tuning of the ELLF consists in obtaining the set of parameters that minimize a χ2 estimator, starting from experimental values reported in the literature [28] for the α coefficient [27]. For SQ20B, the optimal parameters of the ELLF were found to be h = 84,246.7 Gy, z_0_ = 16,279.7 Gy and sigma = 165.016 Gy. The average cellular nuclear radius was 7.9 μm and the global beta was 0.01875 Gy^−2^.

### 2.4 Biodose Actor and surviving fraction predictions

In GATE, ‘actors’ are tools that enable the user to interact with the simulation. It can be used to know the dose deposited in a specific volume of interest. Recently a new actor was developed in the GATE platform to predict the biological dose: the Biodose Actor [22]. This new tool was used in this study to determine the surviving fraction of irradiated cells as predicted by the NanOx radiobiological model. To do so, a dedicated database was generated where precalculated α and β values corresponding to a range of kinetic energies were listed. This was done using the NanOx model for the SQ20B cell line for monoenergetic protons and helium ions with energies ranging from 0.1 MeV/u to 200 MeV/u and 400 MeV/u, respectively.

In practice, calculation of the surviving fraction was computed by using α_mix_ and β_mix_ values, obtained from the Biodose Actor, in the following equation:

Surviving Fraction at dose D = $e^{-\alpha_{\mathrm{mix}}.D-\beta_{\mathrm{mix}}.D^{2}}$

### 2.5 Cell culture and irradiation procedure

The SQ20B cell line was selected for this experiment because it is a well-characterized radioresistant HNSCC model. Such tumors represent a clinically relevant indication for particle therapy and SQ20B has previously been used for investigations of high-LET radiation responses and radiobiological model development [29], [30], [31], [32]. The detailed protocol of the actions undertaken for the cell culture as well as post-irradiation is described in the following article [29]. Briefly, sixteen hours before irradiation, 2.10^5^ SQ20B cells were seeded in monolayer in wells of a 24-well plate having an Aclar bottom. Wells were irradiated independently at doses ranging from 1 to 4 Gy. Three blocks of PMMA of thicknesses 500, 700 and 840 μm ±5% were alternatively inserted in the beam to shift the SOBP and irradiate the cells in three positions, respectively the proximal edge, the plateau and the distal edge (Supplementary Material C). Positioning of the wells was performed by using a motorized device that moved the samples along the horizontal and vertical axes orthogonally to the beam. A laser reference system was used to align the well before the start of the irradiation. Cell survival was calculated using the following equation:

$$\mathrm{SF}=\frac{\text{number of colonies counted}}{\mathrm{PE}\;x\;\text{number of cells seeded}}$$

Where PE (Plating Efficiency) = $\frac{\mathrm{number of colonies counted}\;\mathrm{at}\;0\;\mathrm{Gy}}{\mathrm{number of cells seeded}\;\mathrm{at}\;0\;\mathrm{Gy}.}$

### 2.6 Statistical analysis

Small differences in dose values prevented us from calculating the average surviving fractions across experiments. To overcome this, we fitted each of the four experimental datasets separately according to the linear-quadratic model. Then, the average of the four fitted curves was calculated and plotted. The deviations between this curve and NanOx predictions were calculated in the three irradiation positions by calculating the square root of the average across doses of the following formula: [2*(Sexp-Snanox)/(Sexp+Snanox)]^2^, with Sexp being the experimental surviving fraction and Snanox the one resulting from NanOx predictions.

### 2.7 RBE estimation

RBE was defined as the ratio of dose for photons and the dose for helium irradiation corresponding to a 10% surviving fraction. The dose for photons was extracted using the linear quadratic model (α_X_ = 0.16 Gy^−1^and β_X_ = 0.012 Gy^−2^ [33]. Regarding helium irradiation, the cell survival curve considered was the average of fits of individual experimental datasets, while theoretical RBE values were calculated by using the curve obtained from the NanOx model. As Monte Carlo simulation is a stochastic process, this induces statistical uncertainties in theoretical predictions for which the estimations are detailed in Supplementary Material D.

## 3 Results

The simulated SOBP exhibited a plateau defined as the region receiving at least 90% of the maximum dose extending from 400 μm to 1200 μm depth (Supplementary Material C).

**Fig. 3:**
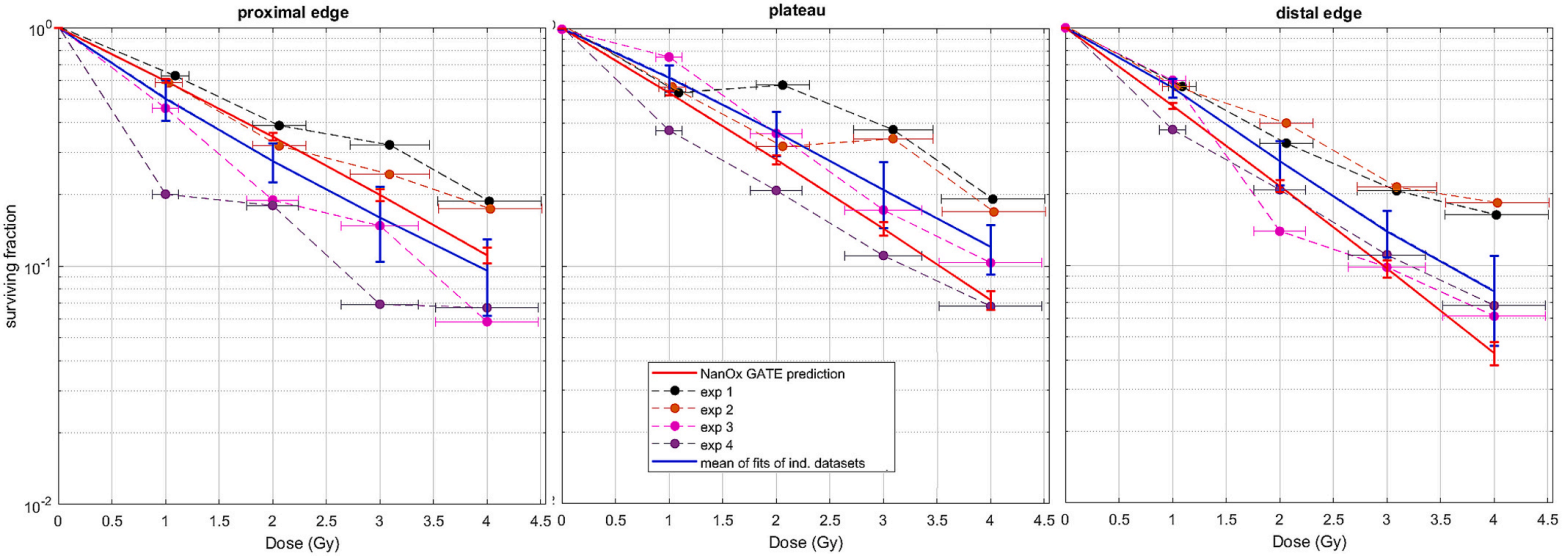
Surviving fraction as predicted by Monte Carlo simulations by the NanOx radiobiological model and experimental data for SQ20B cells irradiated in a helium SOBP. Dashed lines are shown only to distinguish the individual datasets. Error bars represent the standard error of the mean.

The surviving fraction of SQ20B cells, as predicted in GATE by NanOx, decreases as we move towards the distal edge of the SOBP with a surviving fraction at 4 Gy of 11%, 7% and 4% at the proximal edge, plateau and distal edge, respectively (Fig. 3). Experimental data however do not clearly follow this trend with a surviving fraction at 2 Gy ranging from 18% to 39%, 21% to 51% and 14% to 39% in these three irradiation scenarios. When irradiated to 4 Gy the surviving fractions of SQ20B cells were lower and had a smaller range but remained similar for the different irradiation configurations. In particular, the surviving fractions ranged from 6% to 19% at the proximal edge of the SOBP, from 7 to 18% in the plateau and 5 to 18% at the distal edge. Based on the average of the fitted survival curves, the surviving fractions at 4 Gy were 10%, 12%, and 8% at the proximal edge, plateau, and distal edge of the SOBP, respectively (Fig. 3).

The deviations between the average fitted experimental curves and the NanOx predictions were 20%, 35% and 37% for the proximal edge, plateau and distal edge, respectively.

**Fig. 4:**
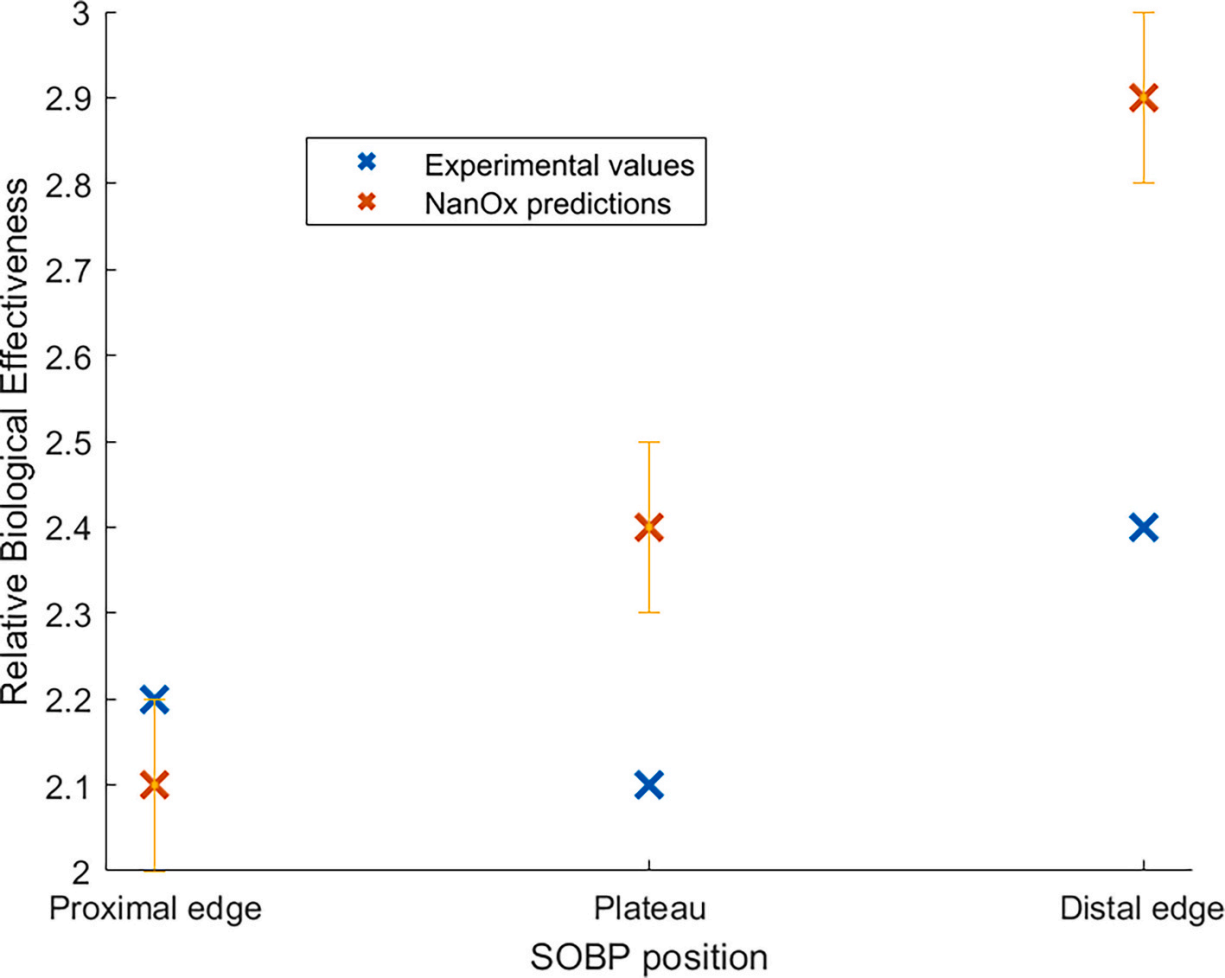
Experimental and theoretical RBE values as predicted by the NanOx model for the irradiation positions.

For SQ20B cells, experimental RBE values were found to be 2.2, 2.1 and 2.4 at the proximal edge, plateau and distal edge of the SOBP, respectively. As for theoretical RBE values derived from the NanOx model, they were 2.1 ± 0.1, 2.4 ± 0.1 and 2.9 ± 0.1 in the three scenarios mentioned (Fig. 4).

## 4 Discussion

In the present study, the surviving fractions of radioresistant SQ20B cells irradiated at three positions along a helium SOBP were measured and compared with predictions from the NanOx model implemented in GATE. NanOx predicted decreasing survival towards the distal edge of the SOBP, whereas this trend was not clearly observed experimentally. Experimental RBE values ranged from 2.1 to 2.4, while NanOx predicted values ranging from 2.1 to 2.9. These results provide additional data on the response of SQ20B cells to helium irradiation in mixed-field conditions and contribute to the ongoing validation of NanOx under the experimental conditions investigated in this study.

NanOx predictions showed deviations ranging from 20% to 37% compared to experimental measurements depending on SOBP position. While the agreement between NanOx predictions and experimental measurements was closest between 0 and 2 Gy, the model tends to systematically underestimate survival at higher doses and in distal SOBP positions, indicating a potential model bias under high-LET conditions. This may reflect limitations in the model parameterization for helium ions, which remains largely constrained by indirect calibration data and limited helium-specific radiobiological measurements. In addition, differences in DNA damage complexity and repair kinetics at higher LET may not be fully captured by the current model formalism, potentially contributing to the observed discrepancy. The NanOx model predicted lower surviving fractions for more distal positions, which is consistent with the idea that higher LET is found in the distal part of the SOBP and is associated with a higher lethality. However, this trend visible in the predictions is not apparent in the experimental measurements, partly because of the large inter-experiment variations which prevent a clear trend in our data from standing out. This disparity may reflect a combination of technical variability, including differences in cell seeding and handling, and intrinsic cellular heterogeneity. Variations in the proportion of subpopulations with higher clonogenic potential, including potentially stem cells, may also contribute to differences in radioresistance [34]. Further investigations are required to clarify this aspect.

Several studies investigating the biological efficacy of helium irradiation on clonogenic assays were conducted. In one such study, human pancreatic cancer cell lines were irradiated at HIT in a SOBP using raster scanning. The experimental RBE was found to range from 1.0 to 1.7 with doses ranging from 1 to 3 Gy [35]. Another study conducted at HIT found for two lung cancer cell lines, an RBE ranging from 1 to 4.3 depending on dose-mean lineal energy and cell line [36]. Separate in vitro work irradiated three human radio-resistant malignant cell lines and found an RBE that rose monotonically with LET increase, consistent with trends between protons and carbon ions [37]. Another study by Kase et al., in which Human Salivary Gland cells were irradiated in several positions of a 6 cm helium SOBP in passive scattering, found an RBE that increases from 1.7 to 2.8 in the last three millimetres of the SOBP [38]. It is unclear whether adding an extra-millimetre to the most distal measurement would result in a further RBE increase. Testing this hypothesis would require reproducing this experiment with more datapoints acquired with sub-millimetre accuracy. To overcome limitations in existing helium beam access, we developed a dedicated pre-clinical beamline focussing on the most distal region of the SOBP and capable of targeting biological samples with sub-millimetre accuracy. This aligns with Mairani et al.'s roadmap call for expanding research infrastructure and facility development tailored to pre-clinical biology studies with helium irradiations [39].

Finally, the relatively low beam energy used in this study (16.9 MeV/u) is below typical clinical energies for helium ion therapy. While this reflects the constraints of a pre-clinical experimental setup, it may limit the direct translatability of the results to clinical treatment conditions. In addition, the present work was restricted to a single radioresistant HNSCC cell line, characterized by mutated p53 and HPV-negative status. Consequently, the present study does not capture the inter- and intra-tumour biological heterogeneity known to influence radiation response, and the findings cannot be assumed to represent the response of other tumour models to helium irradiation. Broader conclusions regarding helium RBE and the predictive performance of NanOx will require validation across a wider range of biological models. Future studies should include additional HNSCC cell lines with different radiosensitivities and molecular characteristics, including distinct HPV and p53 statuses, as well as stem-like cell populations known to contribute to radioresistance [34]. Further investigations may also be relevant in glioma models and other tumour entities such as pancreatic and lung tumors or selected pediatric solid tumors, where the favorable physical characteristics of helium ions could provide a clinical advantage [39]. In particular, glioma models are of interest as survival data following ion irradiation are already available for several cell lines [40], providing a valuable basis for further validation of NanOx predictions under high-LET and mixed-field irradiation conditions. Such a multi-cell-line and multi-tumour-entity approach would not only strengthen the validation of NanOx but also improve the characterization of biological variability in clonogenic response under high-LET and mixed-field irradiation conditions. This broader validation framework will be necessary to determine the extent to which the trends observed in the present SQ20B model can be generalized to other biological settings. Furthermore, the statistical analysis is further limited by the small number of experimental replicates (*n* = 4), which restricts the robustness of uncertainty estimation and prevents the use of formal significance testing.

The present study also demonstrates that the Biodose Actor recently developed in the simulation platform GATE [41], can be used to integrate radiobiological modelling into Monte Carlo simulations, although further validation across additional cell lines and irradiation conditions is required.

In conclusion, this study contributes to the characterization of the biological effect of helium ion irradiation in mixed field conditions in particular on SQ20B cells which may be an indication for future helium ion beam treatment. Differences between NanOx predictions and experimental measurements ranged from 20% to 37% depending on SOBP position. Further validation in additional cell lines and helium irradiation configurations will be required to determine the broader applicability of the model.

## CRediT authorship contribution statement

**Thomas Berger:** Writing – original draft, Visualization, Software, Methodology, Investigation, Formal analysis, Data curation. **Gersende Alphonse:** Writing – review & editing, Visualization, Resources, Methodology, Investigation, Data curation. **Mario Alcocer-Avila:** Writing – review & editing, Software, Data curation. **Caterina Monini:** Writing – review & editing, Software, Data curation. **Charbel Koumeir:** Writing – review & editing, Supervision, Resources, Methodology, Investigation, Data curation, Conceptualization. **Claire Rodriguez-Lafrasse:** Writing – review & editing, Supervision, Resources, Funding acquisition. **Flavien Ralite:** Writing – review & editing, Resources, Investigation. **Nicolas Varmenot:** Writing – review & editing, Resources, Investigation. **Noel Servagent:** Writing – review & editing, Resources, Investigation. **Vincent Métivier:** Writing – review & editing, Resources, Investigation. **Ferid Haddad:** Writing – review & editing, Funding acquisition. **Alexis Pereda:** Writing – review & editing, Software, Resources. **Lydia Maigne:** Writing – review & editing, Software, Resources. **Jean Michel Létang:** Writing – review & editing, Software. **Étienne Testa:** Writing – review & editing, Supervision, Software, Formal analysis. **Michaël Beuve:** Writing – review & editing, Supervision, Software, Resources, Project administration, Methodology, Investigation, Funding acquisition, Formal analysis, Conceptualization.

## Declaration of competing interest

The authors declare that they have no known competing financial interests or personal relationships that could have appeared to influence the work reported in this paper.

## Appendix A Supplementary data

Supplementary material **Supplementary Material A:** Checklist from the RECORDS guidelines listing relevant items that should be detailed in studies involving Monte Carlo simulations. **Supplementary Material B:** Characteristics of the sectors of the range-modulating wheel used to form the SOBP. **Supplementary Material C:** Spread Out Bragg Peak obtained in a water target with 200,000 helium ions resulting from the GATE Monte Carlo simulation of the beamline used to irradiate SQ20B cells with 16.9 MeV/u helium ions at the ARRONAX cyclotron facility. **Supplementary Material D:** Equations quantifying the statistical uncertainties in theoretical predictions
