# Supplementary material for: Comparison of experimental and radiobiological model-predicted survival of human laryngeal squamous cell carcinoma cells in a helium spread-out Bragg peak
Source: Phys Imaging Radiat Oncol. 2026 Jul 28;40:101047. doi: 10.1016/j.phro.2026.101047 (PMC13476767; doi:10.1016/j.phro.2026.101047)
Supplement: Supplementary file 1 [file mmc1.pdf]

Supplementary Materials

Supplementary Material A:

Checklist from the RECORDS guidelines listing relevant items that should be detailed in studies involving Monte Carlo simulations (Sechopoulos et al. 2017 https://doi.org/10.1002/mp.12702).

Title			
	1	Identification as a Monte Carlo study	Surviving fraction of SQ20B cells in a helium spread out Bragg peak: comparison of experimental data with *Monte Carlo* predictions based on the NanOx model
Methods			
Software	2*	Name of Monte Carlo software used, including a reference to a paper and/or report describing the package, if available. If the selected code is not commonly used for the application of the current study or if it necessitated considerable modification, then justification for why a certain Monte Carlo code was selected	GATE 9.4<br><br>Sarrut D, [...], Roncali E. Advanced Monte Carlo simulations of emission tomography imaging systems with GATE. Phys Med Biol. 2021 May 14;66(10):10.1088/1361-6560/abf276. doi: 10.1088/1361-6560/abf276.<br><br>Sarrut D, [...], Buvat I. A review of the use and potential of the GATE Monte Carlo simulation code for radiation therapy and dosimetry applications. Med Phys. 2014 Jun;41(6):064301. doi: 10.1118/1.4871617.
	3*	Release number and/or release date of Monte Carlo software used	Version 9.4.1 released on 07/02/2025
	4a*	If the code has been previously validated or is being validated against previous publications, description of the code's validation in similar or at least relevant applications, preferably via references	GATE is based on GEANT4 Monte Carlo simulations which has been validated for the transport of particles. See for instance :<br><br>http://www.opengatecollaboration.org/<br><br>https://geant4.web.cern.ch/collaboration/<br><br>The biological aspects of our study has been the topic of a previous study for the first implementation:<br><br>Ali Y, Monini C, Russeil E, Létang JM, Testa E, Maigne L, Beuve M. Estimate of the Biological Dose in Hadrontherapy Using GATE. Cancers. 2022; 14(7):1667. doi: 10.3390/cancers14071667<br><br>In the above-mentioned article, the BioDoseActor, the tool used in the present study, was implemented in GATE, using the mMKM and NanOx databases of cell survival predictions as input, to estimate, at a voxel scale, biological outcomes when treating a patient. The HIBMC 320 MeV/u carbon-ion beam line was simulated in GATE. The BioDoseActor was then used to compute an estimation of biological dose, relative biological effectiveness (RBE) and the cell survival fraction for the irradiation of the HSG cell line. The authors then tested the implementation for the prediction of cell

<table>
<tr><td rowspan="2"></td><td></td><td></td><td>survival fraction, RBE and biological dose for the HIBMC 320 MeV/u carbon-ion beamline. The authors found satisfying results for the cell surviving fraction. Regarding the prediction of the biological dose, a 10% relative difference between mMKM and NanOx was reported.</td></tr>
<tr><td>4b*</td><td>If code is being validated against experimental measurements, then detailed description of experimental conditions and of simulation study. For the latter, include any assumptions and simplifications of the experimental conditions</td><td>The present implementation of the biological dose calculations has been extensively tested and an article detailing the algorithms and numerical evaluations will be submitted soon.</td></tr>
<tr><td rowspan="2">Hardware</td><td>5a*</td><td>If study is related to Monte Carlo efficiency, specification of CPU/GPU time, compiler information, and system used to perform the simulations, including CPU/GPU model number</td><td>Not applicable</td></tr>
<tr><td>5b*</td><td>Otherwise specification of order of magnitude of CPU/GPU time and CPU/GPU model number</td><td>Not applicable</td></tr>
<tr><td>Geometry</td><td>6</td><td>Description of simulation geometry, using drawings and tables as needed. Provide all relevant dimensions which are not proprietary and non-disclosable. If possible, provide equivalent composition and dimension information in place of non-disclosable proprietary details</td><td>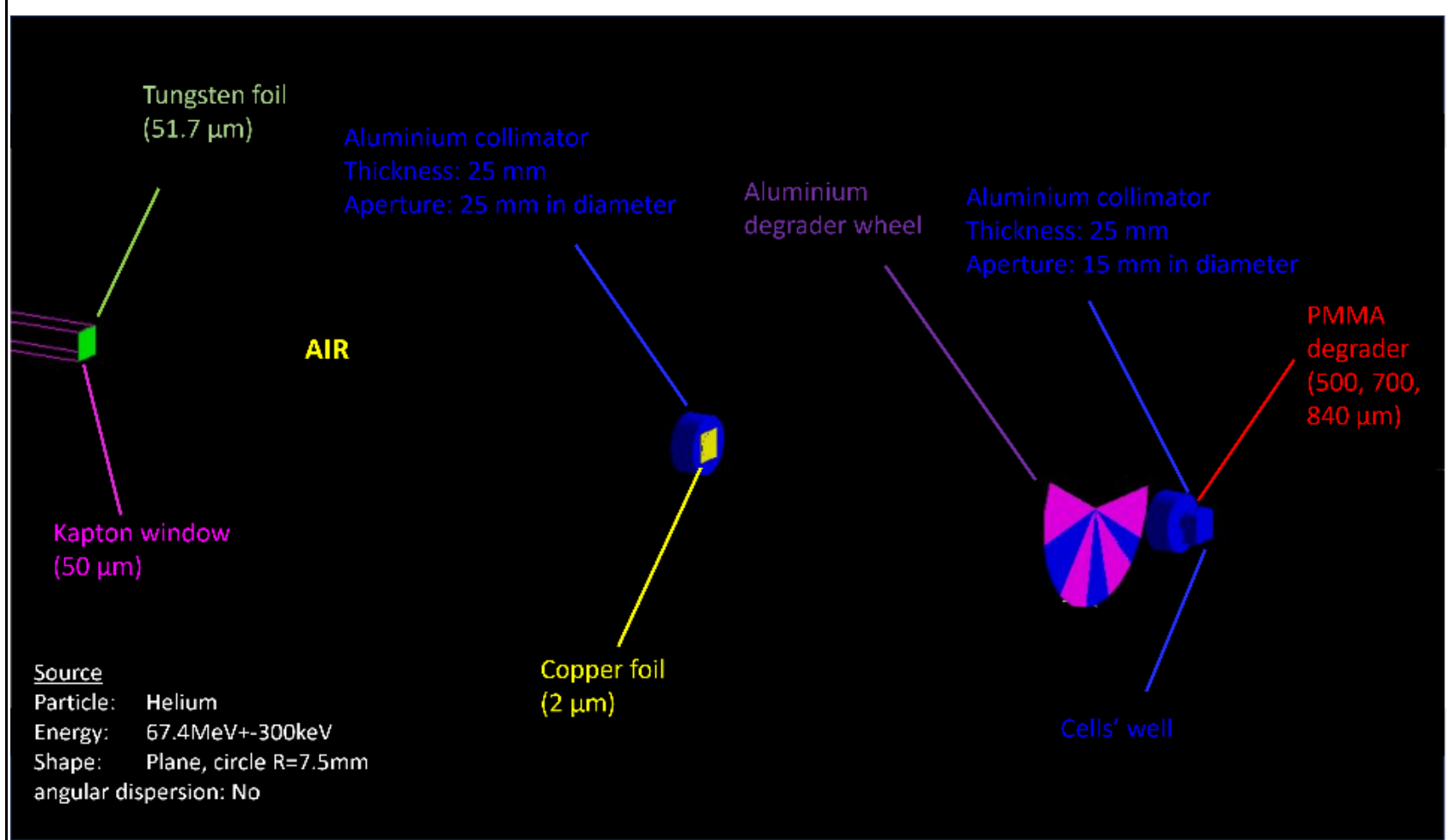
</td></tr>
<tr><td>Materials</td><td>7</td><td>Description of material composition and mass density of each item in the geometry, with references if applicable. Provide the elemental composition and/or, if applicable, the Hounsfield Units conversion method</td><td><table>
<tr><th>Material</th><th>Position</th><th>Thickness</th></tr>
<tr><td>Kapton foil</td><td>-799 mm</td><td>50 µm</td></tr>
<tr><td>Tungsten foil</td><td>-798 mm</td><td>51.7 µm</td></tr>
<tr><td>Collimator - Aluminum</td><td>-84 mm</td><td>25 mm</td></tr>
</table></td></tr>
</table>

Copper foil	-71.45 mm	2 µm
degrader wheel - Aluminum	381 mm	0-350 µm
Collimator Aluminum	471 mm	25 mm
Degrader - PMMA	481 mm	500, 700, 840 µm
Aclar foil (start of cells well) - PCTFE	493.5 mm	25 µm
water detector	501 mm	15 mm

Source	8*	Description of the source, including: source of phase-space files if used; model used to generate source; and model parameter values. Provide reference, if applicable	Source:<br>-Particles: helium<br>-Total kinetic energy: 67.4 MeV +/- 300keV<br>-Shape: Plane, circle, R=7.5 mm<br>-No angular dispersion
	9*	Specification of the cross-section data used along with derived quantities such as stopping powers	The Monte Carlo simulations were run using the QGSP_BIC_EMZ physics list as it accurately models both hadronic and electromagnetic interactions in our energy range. More details are available here: https://geant4-userdoc.web.cern.ch/UsersGuides/PhysicsListGuide/html/index.html
Physics and transport	10*	Specification of relevant transport parameters used, such as energy and particle weight cutoffs, step sizes and thresholds. Also transport algorithms if there is more than one option for the code used. Specifying use of the default is adequate if it is unique and documented by citation	PhysicsList: QGSP_BIC_EMZ<br>setEMin: 0.01 keV<br>setDEDXBinning: 350<br>setLambdaBinning: 350<br><br>For the water detector cuts were set to: 2000 m for gammas, electrons and positrons. A step of 1 um was used. In addition, the step limiter was used for all relevant particles.
	11*	Specification of variance reduction (VRT) and approximate efficiency improvement techniques (AEIT) used, and their parameters. References describing them or specific descriptions of any new techniques should be included if they are not included in the Monte Carlo software as built-in, documented options	Not applicable

Scoring	12*	Specification of the relevant scored quantities. Provide tally names if using a standard Monte Carlo software that includes them, but also include what physical quantity is scored by the tally. When results are binned, tabulated values should make clear what the variable means, e.g., the mid-point, top or range of a bin	The Dose Actor as well as the Biodose Actor were used to estimate, respectively, the physical dose and the α_{mix} and β_{mix} in the voxels of the target. α_{mix} and β_{mix} values were then used to calculate the biological dose and the surviving fraction of SQ20B cells.
	13*	Number of histories and number of source particles used. If various simulations involve different numbers of histories and source particles, at least provide range	200,000 to 1,000,000 simulated helium ions per simulation
	14*	Description and references, as appropriate, of the method used to estimate statistical uncertainty, including if estimated by the history-by-history or the batch method	$\sigma^2_{\alpha_{mix}} = \left(\frac{1}{Z}\right)^2 \sigma^2_{\alpha_{mix}Z} + \left(\frac{\alpha_{mix}Z}{Z^2}\right)^2 \sigma^2_Z + 2\,\frac{\alpha_{mix}Z}{Z^3}\,\sigma_{\alpha_{mix}Z,Z}$<br>$\sigma^2_{\sqrt{\beta_{mix}}} = \left(\frac{1}{Z}\right)^2 \sigma^2_{\sqrt{\beta_{mix}}Z} + \left(\frac{\sqrt{\beta_{mix}}Z}{Z^2}\right)^2 \sigma^2_Z + 2\,\frac{\sqrt{\beta_{mix}}Z}{Z^3}\,\sigma_{\sqrt{\beta_{mix}}Z,Z}$<br>$\frac{\partial D_{bio}}{\partial \alpha_{mix}Z} = \frac{s}{\sqrt{\alpha^2_{ref} + 4\,\beta_{ref}\,\left(s\,\alpha_{mix}Z + s^2\,(\sqrt{\beta_{mix}}Z)^2\right)}},$<br>$\frac{\partial D_{bio}}{\partial \sqrt{\beta_{mix}}Z} = \frac{2\,\sqrt{\beta_{mix}}Z s^2}{\sqrt{\alpha^2_{ref} + 4\,\beta_{ref}\,\left(s\,\alpha_{mix}Z + s^2\,(\sqrt{\beta_{mix}}Z)^2\right)}},$
Analysis	15*	Description of how scored quantities are normalized and/or converted to other metrics. List physical conversion factors used and provide references, if applicable, if there are multiple values for these in the literature	The Dose Actor and the Biodose Actor were used to quantify the physical dose and the α_{mix} and β_{mix} values in each voxels of the target.<br>The surviving fraction of SQ20B cells was then calculated using the following formula:<br>Surviving Fraction at dose D = $e^{-\alpha_{mix}.D-\beta_{mix}.D^2}$
	16*	Description of how scored quantities are de-noised or otherwise filtered, with references, if applicable. If	Not Applicable

		none, then this should be specifically mentioned	
Results			
	17*	Results of validation, unless code has been previously validated	The aim of the present study was not to validate a Monte Carlo code.
	18*	Scored quantities with statistical uncertainty including a specification of the confidence limits used. In general, graphical representation of results such as depth-dose curves or spectra should be histogram rather than point plots, and include uncertainty estimates with error bars in the graphs or text in the caption	Error bars, showing the statistical uncertainty of surviving fraction ranging from 2% to 11% are plotted in Figure 2, based on the equation detailed in Supplementary Material C. Uncertainty on theoretical RBE was also quantified and added to the manuscript.
Discussion			
	19	Discussion of study limitations, including sources of potential bias, statistical uncertainty, and generalizability	Statistical uncertainty has been quantified and controlled and do not appear as a strong limitation. Therefore, the main limitations are those described in the next box.
	20	Discussion of assumptions and approximations and their potential effect on the results, given the knowledge gained	There are several factors that may limit the accuracy of the estimations:<br>-The one associated with the GEANT4 simulation<br>-The one associated with the predictions by the NanOx model and the determination of the alpha, beta coefficients from the predicted cell survival curves;<br>-The one associated with the approximation introduced by Kanai necessary to calculate the α_{mix} and β_{mix}

Supplementary Material B: Characteristics of the sectors of the range-modulating wheel used to form the SOBP.

The thickness of each sector causes a specific reduction in kinetic energy of the incident ions (total kinetic energy: 67.4 MeV) which reduces the range of the corresponding pristine Bragg peaks. The superimposition of these Bragg peaks then forms an SOBP. The relative weight of each pristine Bragg peak defines the angle span of each sector and was determined by using the Non-Negative Least Square algorithm to obtain a uniform dose in the SOBP.

Sector number	Degrader's aluminium thickness (μm)	Weights (%)
1	0	41.98
2	50	10.37
3	100	10.86
4	150	8.89
5	200	6.91
6	250	7.9
7	300	4.69
8	350	8.4

Supplementary Material C: Spread Out Bragg Peak obtained in a water target with 200,000 helium ions resulting from the GATE Monte Carlo simulation of the beamline used to irradiate SQ20B cells with 16.9 MeV/n helium ions at the ARRONAX cyclotron facility.

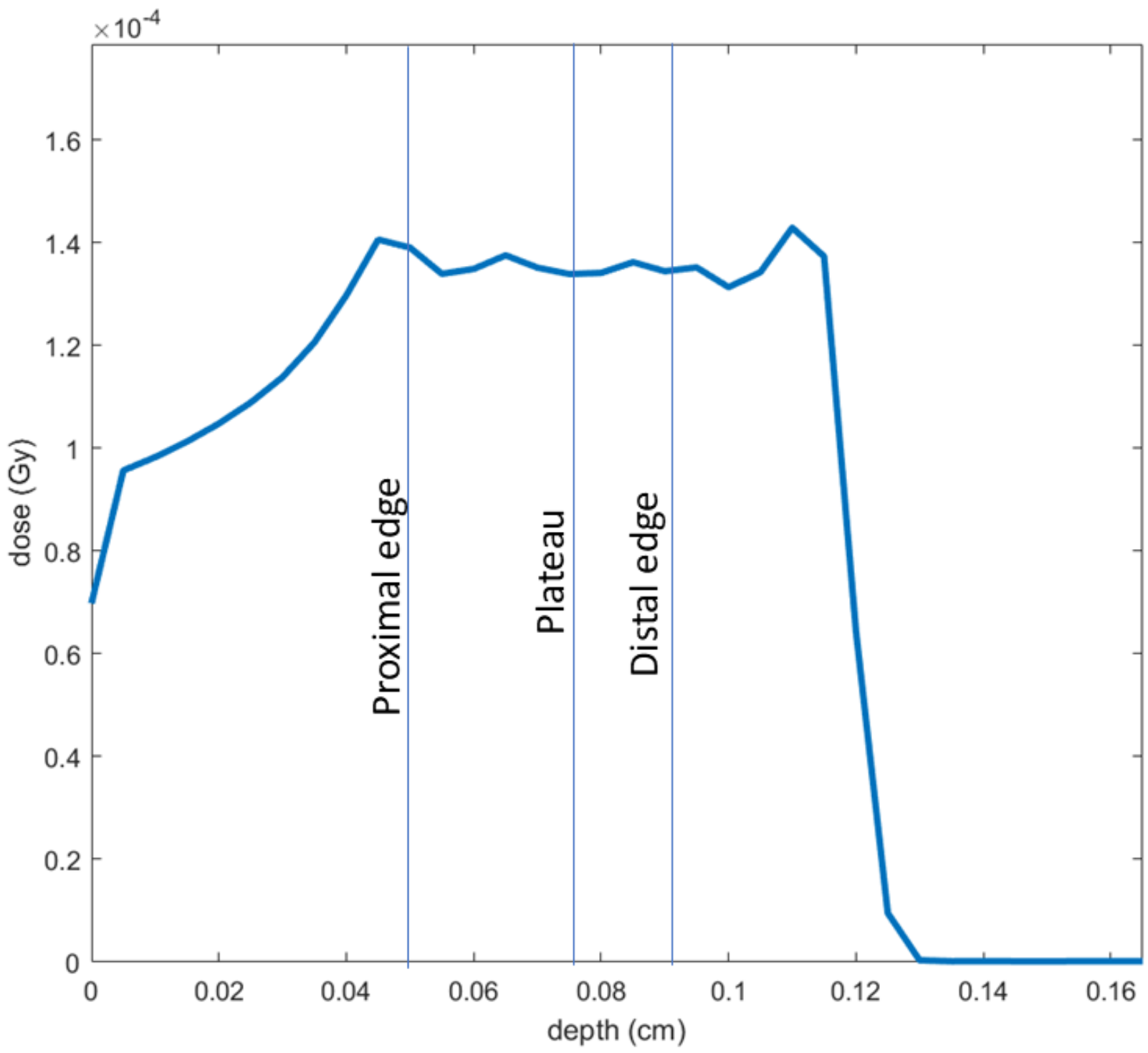

Supplementary Material D: Equations quantifying the statistical uncertainties in theoretical predictions

Relative uncertainty on surviving fraction (used in Figure 2):

$$\frac{\Delta S}{S} = \sqrt{(\alpha_{mix}.D)^2(\frac{\Delta\alpha_{mix}}{\alpha_{mix}})^2 + (\beta_{mix}.D^2)^2(\frac{\Delta\beta_{mix}}{\beta_{mix}})^2}$$

RBE relative uncertainty at 10% surviving fraction:

To determine the dose corresponding to a 10% surviving fraction, we write:

$$-\alpha_{mix}D_{10\%mix} - \beta_{mix}D_{10\%mix}{}^2 = \ln(S_{10\%}) = \ln(0.1)$$

Then we define $\Delta = \alpha_{mix}{}^2 - 4.\beta_{mix}.\ln(0.1)$

Then, $D_{10\%mix} = \frac{-\alpha_{mix}+\sqrt{\Delta}}{2.\beta_{mix}}$

The uncertainty on $D_{10\%}$ is then calculated with the following equation:

$$(\Delta D_{10\%mix})^2 = (\Delta\alpha_{mix})^2.\left(\frac{-1}{2\beta_{mix}} + \frac{1}{2\sqrt{\Delta}}.\frac{2\alpha_{mix}}{2\beta_{mix}}\right)^2 + (\Delta\beta_{mix})^2.(\frac{\frac{-4\ln(0.1)\,.\,2\beta_{mix}}{2\sqrt{\Delta}} - 2(-\alpha_{mix} + \sqrt{\Delta})}{4\beta_{mix}{}^2})^2$$

Based on this result, the uncertainty of the RBE is then defined as:

$$(\frac{\Delta RBE}{RBE})^2 = (\frac{\Delta D_{10\%\,\gamma}}{D_{10\%\,\gamma}})^2 + (\frac{\Delta D_{10\%\,mix}}{D_{10\%\,mix}})^2$$

Which can be approximated, assuming only statistical uncertainty as

$$(\frac{\Delta RBE}{RBE})^2 = (\frac{\Delta D_{10\%\,mix}}{D_{10\%\,mix}})^2$$
